# The Feasibility of Applying Artificial Intelligence to Gastrointestinal Endoscopy to Improve the Detection Rate of Early Gastric Cancer Screening

**DOI:** 10.3389/fmed.2022.886853

**Published:** 2022-05-16

**Authors:** Xin-yu Fu, Xin-li Mao, Ya-hong Chen, Ning-ning You, Ya-qi Song, Li-hui Zhang, Yue Cai, Xing-nan Ye, Li-ping Ye, Shao-wei Li

**Affiliations:** ^1^Taizhou Hospital of Zhejiang Province Affiliated to Wenzhou Medical University, Linhai, China; ^2^Key Laboratory of Minimally Invasive Techniques and Rapid Rehabilitation of Digestive System Tumor of Zhejiang Province, Taizhou Hospital Affiliated to Wenzhou Medical University, Linhai, China; ^3^Department of Gastroenterology, Taizhou Hospital of Zhejiang Province Affiliated to Wenzhou Medical University, Linhai, China; ^4^Institute of Digestive Disease, Taizhou Hospital of Zhejiang Province Affiliated to Wenzhou Medical University, Linhai, China; ^5^Health Management Center, Taizhou Hospital of Zhejiang Province Affiliated to Wenzhou Medical University, Linhai, China; ^6^Taizhou Hospital, Zhejiang University, Linhai, China; ^7^Department of Gastroenterology, Renmin Hospital of Wuhan University, Wuhan, China; ^8^Taizhou Hospital of Zhejiang Province, Shaoxing University, Linhai, China

**Keywords:** artificial intelligence, early gastric cancer, screening, improving, application

## Abstract

Convolutional neural networks in the field of artificial intelligence show great potential in image recognition. It assisted endoscopy to improve the detection rate of early gastric cancer. The 5-year survival rate for advanced gastric cancer is less than 30%, while the 5-year survival rate for early gastric cancer is more than 90%. Therefore, earlier screening for gastric cancer can lead to a better prognosis. However, the detection rate of early gastric cancer in China has been extremely low due to many factors, such as the presence of gastric cancer without obvious symptoms, difficulty identifying lesions by the naked eye, and a lack of experience among endoscopists. The introduction of artificial intelligence can help mitigate these shortcomings and greatly improve the accuracy of screening. According to relevant reports, the sensitivity and accuracy of artificial intelligence trained on deep cirrocumulus neural networks are better than those of endoscopists, and evaluations also take less time, which can greatly reduce the burden on endoscopists. In addition, artificial intelligence can also perform real-time detection and feedback on the inspection process of the endoscopist to standardize the operation of the endoscopist. AI has also shown great potential in training novice endoscopists. With the maturity of AI technology, AI has the ability to improve the detection rate of early gastric cancer in China and reduce the death rate of gastric cancer related diseases in China.

## Introduction

Gastric cancer (GC) is the fifth-most common malignant tumor and the third leading cause of cancer-related death in the world (1, 2). Gastric cancer is also the second leading cause of cancer deaths in China, with a standardized 5-year survival rate of only 27.4% (3). According to related research, there were approximately 1 million newly diagnosed gastric cancer cases in 2008, 47% of which were in China, which accounted for half of the global gastric cancer deaths (4, 5). Of note, however: the 5-year survival rate of early gastric cancer (EGC) was over 90%, which was much higher than that of advanced gastric cancer (AGC) (30%) (6–8). Therefore, improving the detection rate of endoscopic EGC is essential for reducing the mortality, labor loss, and tumor treatment cost caused by GC (9).

The diagnosis of EGC is related to the ability of endoscopists to adequately analyze endoscopic images, a skill cultivated through extensive training over a long period (10). While the diagnostic level of EGC has gradually improved in China with the establishment and improvement of many endoscopic centers, the rate of endoscopy diagnoses differs among regions, and areas with better economic and medical development consequently have better equipment and training systems, whereas facilities in remote areas tend to have insufficient training in endoscopy technology and a lack of experience endoscopists (11). Therefore, it is necessary to improve the detection rate of EGC under endoscopy with instrument-assisted diagnostic tools, especially in areas where there is a shortage of endoscopists.

With the rapid development of computer science and technology, artificial intelligence (AI) technology is maturing, allowing it to be used to improve accuracy in a variety of medical situations (12). The number of endoscopists in China is insufficient at present, being primarily concentrated in the top three hospitals. Most community hospitals lack the proper equipment for endoscopy, and even in cases where they do have the equipment, operators are lacking. Community hospitals are unable to receive diverted patients, resulting in a heavy burden on endoscopists in tertiary hospitals. Under this massive workload, endoscopists struggle to accurately identify any lesions, and EGC is even more difficult to detect. Therefore, to resolve the current situation, attention has been focused on the feasibility of applying AI technology to endoscopy (11).

Among AI technologies, neural networks, represented by cirrocumulus neural networks, have demonstrated remarkable progress, achieving feats comparable to or even surpassing human beings in the field of image recognition. AI is not affected by subjectivity, fatigue, experience, or other factors. It performs medical image-assisted diagnoses well and has a high focus recognition rate. In addition, its learning ability is continuous and improves with increasing exposure to training data. AI has shown great potential in endoscopy, including in screening for EGC (12).

## Analysis of Recent Trends in the Literature on Artificial Intelligence and Early Gastric Cancer

**FIGURE 1:**
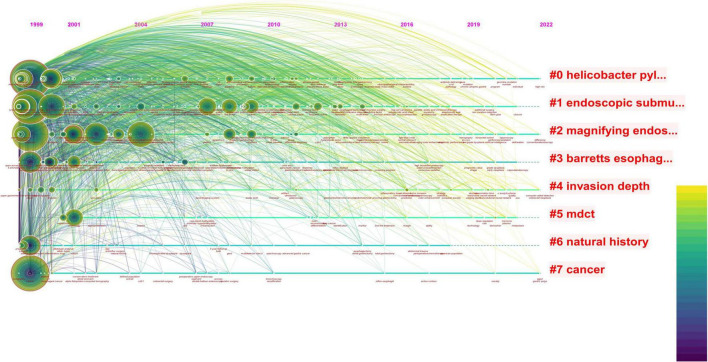
The red font in the figure represents the keywords with the highest frequency in the included literature, the circle represents the articles published in that year, the size represents the number of articles published, and the color of the line represents the year. The reports on endoscopy and EGC were mainly concentrated between 1999 and 2010, with less and less relevant literature published in this field after that point. In the last decade, the topic of combining endoscopy with EGC has no longer been a research topic of interest.

**FIGURE 2:**
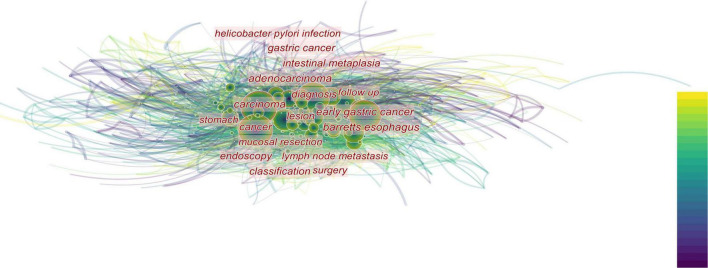
The closer the font is to the center of the figure, the more attention is paid. In addition, the size of the circle indicates the number of relevant publications. The top-down color indicates the year. The diagnosis gets the most attention, followed by the various digestive diseases that surround the diagnosis.

**FIGURE 3:**
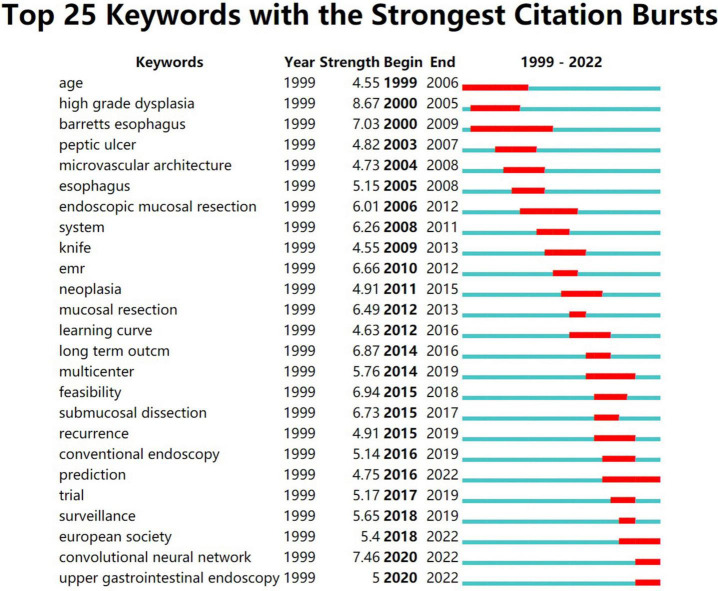
This figure shows the 25 keywords with the highest frequency in the literature and the attention paid to these keywords over time. It’s not hard to see that convolutional neural networks are beginning to attract attention.

We analyze current research trends in AI, EGC, and endoscopy by searching relevant topics in the Web of Science core database. The analysis results are presented with citeSpace drawings. In our search concerning endoscopy and EGC, we found 1,664 related articles, and 1,625 were used in the final analysis. In our search concerning endoscopy and AI, we found 392 related articles, and 354 were used in the final analysis. In our search concerning AI and EGC, we found 67 relevant articles, and 58 were used in the final analysis. We then combined these three search terms to perform retrieval again and found 55 relevant articles, and 40 were used in the final analysis. We analyzed the topics related to endoscopy and EGC, obtaining three figures (Figures 1–3). On analyzing these three figures, we found that the studies on endoscopy and EGC were mainly concentrated between 1999 and 2010, without much research or attention focused on these topics in the last decade. In line with Figure 3, we also found that various endoscopic operation techniques have been attracting increasing attention in recent years. In addition, we found that convolutional neural networks have received a lot of attention in the last 3 years.

**FIGURE 4:**
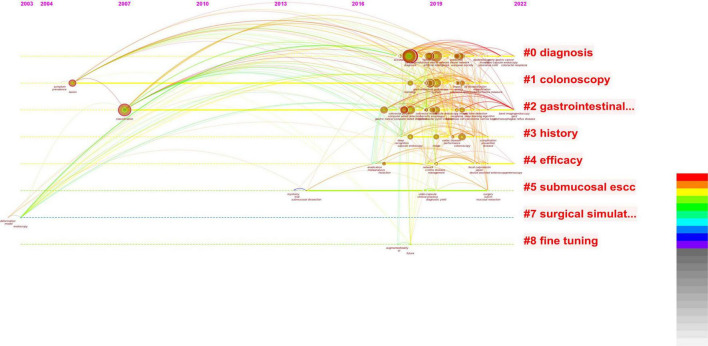
Based on combined images, the studies related to AI are concentrated in the last 3 years, and there is an obvious growth trend.

**FIGURE 5:**
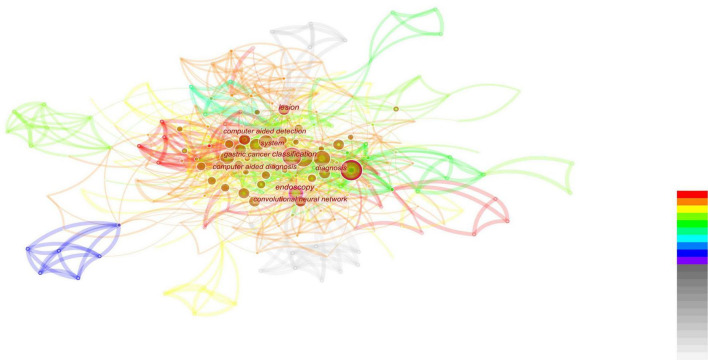
This figure is based on the literature analysis of artificial intelligence and endoscopy. It can be seen that the research hotspots under this topic are the classification of gastric cancer and computer-aided examination and diagnosis. At the same time, convolutional neural networks also appear in hot spots, indicating that convolutional neural networks are showing an increasing trend in the application of artificial intelligence.

**FIGURE 6:**
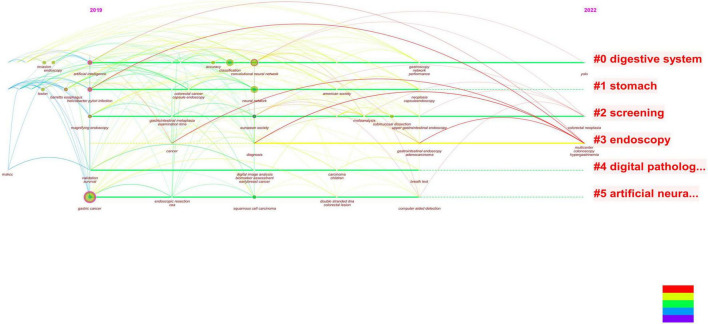
By analyzing the figure, it can be seen that when combined with literature on early gastric cancer and artificial intelligence, both of these topics have occurred in recent years. Additionally, this year’s study focused on endoscopic screening.

**FIGURE 7:**
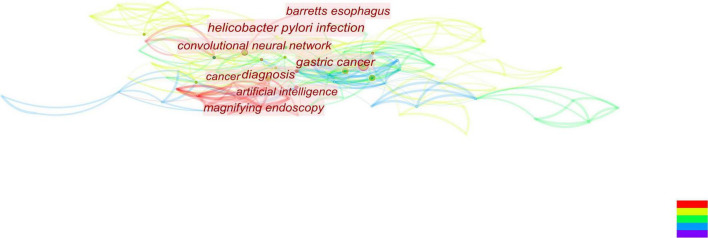
According to this figure, we can see that the convolutional neural network is currently attracting a lot of attention and is closely associated with gastric cancer.

**FIGURE 8:**
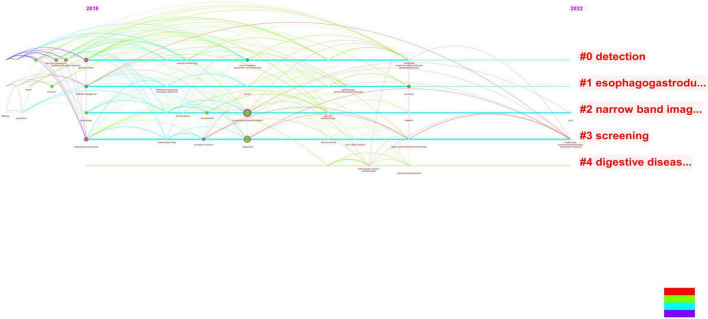
It can be seen from the revised figure that the literature studies on the combination of early gastric cancer, artificial intelligence, and endoscopy have taken place in recent 3 years, and the main research hotspots in 2022 are focused on screening, i.e., applying artificial intelligence to endoscopy to screen early gastric cancer.

**FIGURE 9:**
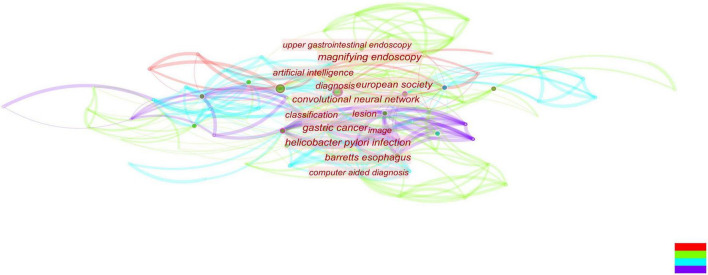
Combined with literature on early gastric cancer, artificial intelligence, and endoscopy, convolutional neural networks occupy the center and become an absolute research hotspot.

We next analyzed the literature on endoscopy and AI and obtained two similar figures (Figures 4, 5). By combining these two pictures, we found that the combination of AI and endoscopy has been a hot topic in the past 3 years, specifically for the detection of early cancer. We then searched for related literature on AI and EGC as well as the combination of these three topics and obtained four figures (Figures 6–9). Based on our analysis of these four figures and in combination with previous findings, we concluded that the application of AI to endoscopy in order to detect EGC remains a hot research topic, although relevant studies are lacking, so further new findings are awaited. By analyzing the existing literature, we also found that current research is focusing on convolutional neural networks and screening.

This review will focus on these three aspects: convolutional neural networks, the dilemmas associated with EGC screening, and the feasibility of applying AI to GC screening.

## The Technology of Artificial Intelligence

Machine learning and deep learning are considered two sub-technologies of AI (13). Deep learning can be used for prediction and judgment (14, 15). Machine learning can automatically improve computer algorithms through experience and use data or past experience to optimize the performance standards of computer programs (13). Both of these are the most commonly used technologies to build AI models (16).

An artificial neural network (ANN) is a monitoring model whose model structure is very similar to that of neurons in the human central nervous system (17, 18). Neurons are joined to create a network as a computational unit. When data enter the input layer, they travel through a series of concealed layers before reaching the output layer (18). Before ANNs can be utilized, they must first be trained, which entails splitting data into “training sets” that define the network structure and “test sets” that assess the ANN’s ability to anticipate the intended output (19, 20).

To meet the need for increased performance, more and more complex neural networks are developed, resulting in the concept of deep learning. Deep learning works by progressively extracting higher-level features from raw input using multi-level structures (21). A deep neural network (DNN) is derived from an ANN and consists of multiple continuous filters that can automatically detect and extract important features of input data (22, 23). To improve performance, a large amount of marked training data is required, which involves a combination of deep learning and reinforcing learning.

**FIGURE 10:**
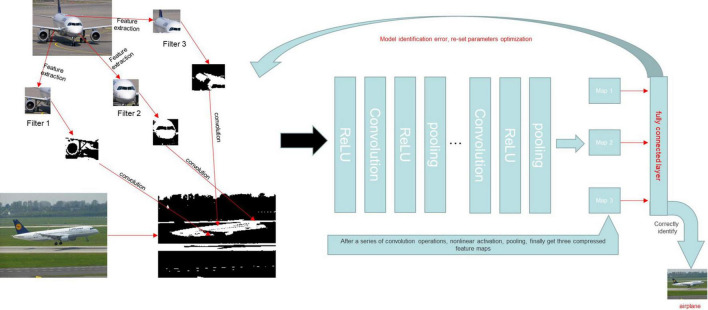
A CNN model was trained to identify whether or not the content of a given picture was an airplane. We assumed that the characteristics of the aircraft were the tail, engine, and fuselage and set the characteristics as the convolution kernel. The image was then converted into a matrix that a computer could recognize. The eigenmatrix of the sample was obtained by a convolution operation between the convolution kernel and the sample image. A non-linear activation function was then used to perform a non-linear activation operation on the eigenmatrix to improve the sparsity of the network and reduce the interdependence of parameters. The pooling layer was used to reduce the dimension of the feature matrix, compress the image features, remove the redundant information, and reduce the amount of calculation. Finally, we converted the calculated eigenspace mapping sample marker space into a one-dimensional vector through the full connection layer to obtain the complete image features. After completing the above steps, we also established an error function to determine the accuracy of the output. The convolution kernel parameters were adjusted to reduce the error and obtain the actual features of aircraft images.

At present, the most widely used and effective network is the convolutional neural network (CNN). It has shown great potential in many fields, such as pathological analyses, computed tomography, and magnetic resonance imaging analyses (23–28). A CNN is a feedforward multi-layer network in which the information flow is unidirectional, i.e., from input to output, and each layer uses a set of convolution kernels to perform multiple transformations in the process of information flow (14). Through this process, information characteristics are extracted. A CNN model mainly includes a convolution layer, pooling layer, and full connection layer. A novel network model is created based on the CNN model by merging multi-layer convolution and multi-layer pooling, which can increase network structure accuracy (22). A traditional CNN is mainly composed of two parts: the multi-component convolution layer and classification layer. The convolution layer’s primary job is to extract features from the input data. When the input data is an image, e.g., and the observed item is an abstract entity, the convolution layer extracts the abstract and valuable texture elements from the image and sends them to the classification layer, which is primarily responsible for classifying the input image (29, 30). Furthermore, because a CNN uses the convolution operation of the weight-sharing scheme, the number of network parameters required by a CNN is dramatically decreased compared to completely linked networks with the same number of network layers, thereby reducing the risk of over-fitting. A CNN may be very profound and complex in the eyes of outsiders, but its working mode is briefly expressed in Figure 10. A CNN is currently being used to solve a variety of computer recognition challenges, including picture categorization, target detection, and image synthesis (31). This model imitates the recognition and the processing of image by the human brain, making the processing of image information faster and more accurate. At the same time, with the continuous iteration and update of the technology, more images can be identified for review. The recognition of medical examination images, including imaging findings, pathological endoscopic images, and endoscopic images (32). A deep CNN was trained using 1,29,450 skin photos to create 2,032 distinct skin disease presentations (33). The model was then put to the test against 21 board-certified dermatologists, who were shown to be equally skilled at telling the difference between keratinocyte cancer and benign seborrheic keratosis, as well as malignant melanoma and benign nevus (33). This example reflects the great potential of CNN-based AI in the field of image recognition.

If AI can be successfully combined with various clinical examinations, it will greatly improve clinical practice. However, at present this is a brand-new field, so further exploration and experimentation are necessary. In recent years, there have been numerous attempts to integrate AI into various medical fields, including endoscopy. The potential utility of this approach in GC screening is discussed below.

## The Dilemma of Screening for Early Gastric Cancer

Early gastric cancer is difficult to detect, as the early symptoms of GC are not obvious, and some patients do not actually show any early symptoms, while elderly people tend to avoid visiting the hospital for regular examinations (34). Patients who wait for obvious symptoms to visit a doctor often present with advanced GC, missing the optimum treatment window.

At present, only two nations have government-funded GC screening programs: Japan and South Korea (35). Despite the high prevalence of GC, the death to morbidity ratio is low in these countries (0.43 in Japan and 0.35 in Korea), indicating the value of population-based screening in high-risk locations (4, 35). Another experiment looking into whether or not early detection of GC reduced medical expenditures discovered that the cost of treating GC rises considerably with the stage of the disease. Early identification of GC and endoscopic submucosal dissection (ESD) can thus significantly reduce GC treatment costs, indicating that early identification of GC is critical for reducing medical expenditures (34).

However, despite the country’s relatively high incidence of GC, China still lacks a countrywide screening program and the only way to identify EGC is through opportunistic screening (36). A domestic study established a mathematical model to analyze the long-term population impact of an endoscopic screening program on the disease burden of GC patients in China. Experiments have shown that 5.53–4.64 million cases and 7,40,000–5.42 million deaths could be prevented over 30 years with different screening coverage and frequency. It is necessary to carry out large-scale screening in China (37). To address this issue, China must step up its efforts in EGC screening.

Endoscopy is the most effective diagnostic method for gastric cancer and can improve the detection rate of EGC (38). Despite the ongoing progress of endoscopic imaging technology, which has improved the detection rate of EGC, there remains a high rate of missed diagnoses, as the ultimate result of endoscopy largely depends on the endoscopist, and both their experience and operation approach will affect this outcome. Studies have shown that a diagnosis was missed in up to 10% of patients who underwent endoscopy recently. Meanwhile, in a recent randomized clinical trial in Japan, the sensitivity of GC was only 75% (39), indicating that the detection of EGC still has room for improvement.

Some studies have shown that the sensitivity of GC detection can be increased by training endoscopists to improve their operational skills and ability to identify lesions (9). In addition, In today’s clinical setting, each endoscopist must perform the same set of procedures on a large number of patients and identify lesions that are difficult to identify with the naked eye in a large number of endoscopic images. This is difficult for any endoscopist, even an experienced one, resulting in a risk of subjective mistakes (40). Prolonged endoscopy has been shown to cause endoscopists to lose focus, reducing the quality of the examination and perhaps leading to a false-negative diagnosis. According to 10 studies involving 3,787 patients undergoing upper gastrointestinal (GI) endoscopy, 11.3% of upper GI tumors were missed 3 years before they were ultimately diagnosed (41). Missed diagnoses also depend on the type and location of GC and are more pronounced in endoscopists with under 10 years of experience than in more experienced individuals. The physical and mental condition of the endoscopist who performs the procedure also strongly influences the rate of missed diagnoses.

Because of the uneven distribution of population and medical resources in China, the situation of endoscopy in China is more serious than that in some developed countries. The huge workload may result in our missed diagnosis rate being significantly higher than theirs. According to the census results of the number of practitioners of digestive endoscopy conducted in China in 2013, there is a huge gap in the number of endoscopists in China. Moreover, their technical level is not equal, and the doctors in economically developed cities have more medical resources and opportunities for intensive training than those in less developed cities. According to the census data, the number of digestive endoscopy physicians per million people in 20 provinces and cities is lower than the national average of 19. There are now only 30,000 endoscopists in China, despite a demand for endoscopy in the hundreds of millions. Such a big gap has left endoscopists in China with a huge workload, forcing them to reduce the examination time per patient in order to improve efficiency and relieve the pressure of work caused by a labor shortage. Studies have shown that the duration set aside for endoscopy and the rate of disease detection are positively correlated (42). Shorter test times mean a higher rate of missed diagnoses.

In addition, compared with other developed countries, the development rates of digestive endoscopy diagnoses and treatment technology in China are still quite low, and standardized training of endoscopists has not yet matured, resulting in a disparity of technical skills among endoscopists across the country. Operator factors significantly influence the outcome of endoscopy. Compared to other nations, China’s current condition has rendered the stability and sensitivity of endoscopy unreliable. In general, there are three dilemmas facing GC screening in China: (1) there is still a big gap between the development of digestive endoscopy technology in China and that of foreign countries and a systematic and standardized training system has not been implemented; (2) there is a serious shortage of endoscopists in China that is unable to meet the current demand for endoscopy in China; and (3) the accuracy of endoscopy cannot be guaranteed.

## Artificial Intelligence in Screening of Early Gastric Cancer

Endoscopy screening for EGC is a difficult and time-consuming procedure, but it should not be taken lightly, as each missed diagnosis may cause patients to lose out on the most effective therapy option. Early identification and therapy are still the most effective treatments for GC. Endoscopists must therefore thoroughly examine each patient. However, humans are not machines, and long-term endoscopic operation can impair endoscopists’ discriminating capacity and impact the examination quality. The involvement of nurses can increase the rate of lesion identification and the quality of endoscopy by acting as a second observer during the procedure (43). As AI technology advances, it will be possible for AI to be involved in internal examinations as a third observer.

Furthermore, an increasing number of studies have proven that trained CNNs can swiftly identify lesions with an accuracy equivalent to that of endoscopists. A research team from Japan created a model of a CNN-based system using a training model of 13,584 gastroscopic images of GC. The total sensitivity of the model reached 92.2%, and it only took 47 s to examine 2,296 detection images (44). CNN can accurately detect images of invasive GC, and the detection rate of lesions above 6 mm in diameter is 98.6%. A similar study, also from Japan, described training a CNN model in a similar way, and the model recognized each image in just 4 ms (45). These studies show that AI can quickly and accurately identify lesions. If this approach can be applied to the clinical setting, it will reduce the pressure on endoscopists.

The Japan Cancer Research Foundation conducted a study comparing the speed and accuracy of endoscopic image recognition by artificial intelligence and endoscopists. Researchers trained a CNN-based model with 13,584 endoscopy images to work with 67 endoscopists to identify 2,940 images from 140 instances (46). The AI was able to recognize each image in about 40 s, while the endoscopist took about 220 times longer to recognize each endoscope image. On comparing the sensitivity, specificity, and positive and negative predictive values, the AI specificity and positive predictive value were found to be lower than those of endoscopists, while the other two values were higher than those of endoscopists (46). Although the AI model in this trial was able to determine whether or not GC was present in the images, the location, and extent of the tumors in the images were not assessed in detail. The number of endoscopists used for the comparison was also small. However, a CNN was compared with several experienced endoscopists who made their evaluations under the same conditions, so the experimental data obtained were still convincing. We have every reason to believe that by increasing the amount of data even further, the identification ability of AI can be rendered extremely close to that of actual endoscopists or even comparable to that of experienced endoscopists.

A similar experiment was carried out in the Department of Gastroenterology at the People’s Hospital of Wuhan University in China. They used an AI system designed by themselves to validate the results using 200 endoscopic pictures. Its accuracy, sensitivity, and specificity for identifying EGC were 92.5, 94, and 91%, respectively, compared to 89.7, 93.9, and 87.3% for experienced endoscopists (47). The Department of Gastroenterology, Peking University People’s Hospital also trained their own CNN model and obtained similar results (48). The above two experiments also compared the AI trained themselves with endoscopists and obtained similar results. However, the AI used in these experiments has common limitations, as it was only able to recognize static endoscopic images, and only a small number of endoscopists were involved. If more endoscopists had been included in the comparison, the comparison reliability would have been higher. In addition, data from a hospital were used in the above tests to analyze the training model, and there was no strict quality control of the endoscopic images. The same problem also appeared in the experiment comparing AI and endoscopists conducted by Drum Tower Hospital affiliated with Nanjing University Medical School. The results showed that AI was superior to endoscopists with regard to accuracy (85.1–91.2%), sensitivity (85.9–95.5%), and specificity (81.7–90.3%). However, it also has its innovation point, which tests the identification ability of the lesions of the intern endoscopists assisted by artificial intelligence, and makes a comparison with the experts. The sensitivity of interns increased from 82.7 to 94.7%, and their performance was comparable to that of specialists (sensitivity: 94.7 vs. 97.4%) (49). A comparative experiment was also conducted in the Sun Yat-sen University Cancer Center, in which endoscopists were innovatively divided into three levels: expert endoscopist (10 years of endoscopy experience), competent endoscopist (5 years of endoscopy experience), and trainee endoscopist (2 years of endoscopy experience); their evaluations were then compared with AI. Experimental data showed that the diagnostic sensitivity of AI was similar to that of endoscopy experts (0.942 and 0.945). The positive predictive value for experts was 0.932, while that for AI was 0.814. In terms of the negative predictive value, AI was slightly better than the expert endoscopist (0.980) and higher than the competent endoscopist (0.951). AI also demonstrated superior capability to trainees, although the positive predictive value was similar between the two. The advantage of this experiment is that the samples were obtained from multiple hospitals, which reduces the error potentially caused by using samples from a single hospital. At the same time, the quality of the endoscope image was strictly controlled. Each endoscope image was manually marked by two experienced endoscopists and any images that did not meet the requirements were eliminated. However, that study also had limitations, such as only using white-light images. In addition, the AI’s training and external validation sets were obtained retrospectively, which may have led to a certain degree of selection bias. In addition, this experiment did not use a specific method to process images obtained at different positions in the same series of videos, which may have caused some inheritance bias (50).

Despite the limitations, that experiment and each of the others described above had their own innovations. At the same time, there are many similar retrospective experiments, all of which have verified the utility of AI in lesion identification and demonstrated the great potential of AI in endoscopy. Based on the above findings, we believe that AI can quickly identify lesions with accuracy, greatly reducing the current burden on endoscopists.

Furthermore, there are many other aspects to AI that bear highlighting. For example, the AI system developed by the People’s Hospital of Wuhan University was able to divide gastroscopic images into 26 anatomical areas with an accuracy of 65.9%, which was comparable to the rate of 63.8% for experienced endoscopists, and reduced the rate of image sites missing by 15% in a comprehensive randomized controlled trial (47). In addition, the authors found that using an AI system in routine endoscopy can dramatically minimize the number of missed locations. As a result, the use of AI is expected to reduce the number of cases of GC missed due to insufficient endoscopy (51).

**FIGURE 11:**
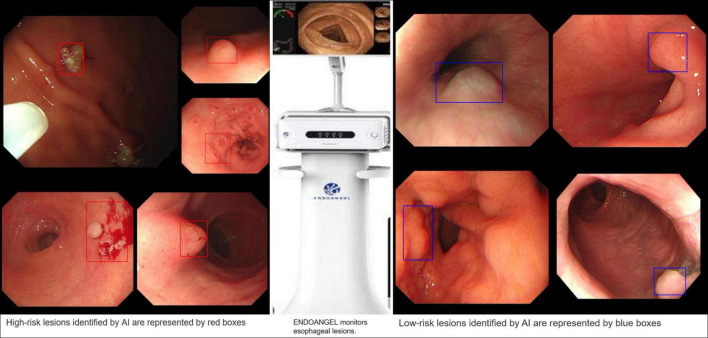
EndoAngel-assisted endoscopy. The image on the left shows high-risk lesions, and the image on the right shows low-risk lesions. During endoscopy, AI automatically identified and evaluated the lesion. If it detected a high-risk lesion, a red prompt box appeared, while a blue prompt box appeared for low-risk lesions. The prompt box not only helps the endoscopist quickly identify the lesion but also helps doctors carry out an accurate sampling biopsy.

A successful case of applying AI to clinical practice was recently reported in the “EndoAngel.” This is an AI quality control auxiliary diagnosis system of digestive endoscopy based on a CNN model that can effectively monitor the blind area on GI imaging, assist in the detection of suspicious lesions in real time, improve the quality of endoscopy and improve the detection rate of GI tumor lesions; in addition, it is also equipped with a scoring training system for upper and lower gastrointestinal endoscopy. It is a fully functional AI product integrating quality control and auxiliary diagnostic functions. The People’s Hospital of Wuhan University has cooperated with 13 other hospitals to conduct a functional verification study of the EndoAngel for the early diagnosis of GC. The EndoAngel has a 92% diagnostic accuracy in EGC, and its main working mode is shown in Figure 11.

The technological skill level of endoscopic physicians is disparate at present, and the operation is not sufficiently standardized, affecting the endoscope quality. The ADM system, based on a CNN and developed by the People’s Hospital of Wuhan University is intended to provide the following statistical quality indicators: colonoscopy time, cecal endoscopy intubation rate (CIR), adequate bowel preparation rate, polyp detection rate (PDR), adenoma detection rate (ADR), gastroscopy time, and gastric precancerous condition (GPC) detection rate. The system may also simultaneously analyze the quality of each endoscope and provide rapid feedback to the operator. Controlled experiments verified that the detection rate of precancerous lesions increased in the endoscopic group with AI feedback (3–7%) as well as in the control group (3.5–3.9%) (11). These findings suggest that quality management of endoscopy operations can significantly increase the screening rate. Furthermore, AI can not only serve as a quality control system to supervise endoscopists’ performance but also participate in the standardized training of endoscopists, reduce the endoscopist training time.

Extensive endoscopy cannot be performed in China at present. One reason for this is a lack of corresponding equipment in community hospitals, and another is the scarcity of endoscopists, with this latter reason being the main issue. Endoscopists are in short supply in China, being mostly centered in major hospitals; this means that even if rural hospitals have similar technology, no one is available to operate them. The advent of AI appears to be a game-changer. In terms of the sensitivity and accuracy of inspections, the present AI model based on a CNN appears to have the equivalent skill to professional endoscopists (52). If AI were to be introduced to community hospitals in China, it would be equivalent to having an experienced endoscopist in each hospital. With this approach, large-scale screening for EGC will also become possible.

## Conclusion

Thus far, retrospective trials of AI screening for early stomach cancer have yielded promising results. The precision and accuracy with which lesions are identified are equivalent to those of endoscopists. If AI were to be employed in the early stomach cancer screening process, it would significantly improve the poor detection rate of EGC in China (53). Furthermore, AI, which is still being developed, can aid in training endoscopists, making endoscopy training in China more unified and uniform. However, while AI has demonstrated significant potential in early stomach cancer screening, such clinical trials are uncommon at present, and there remains much research to complete before AI can be widely used in this regard.

## Author Contributions

All authors listed have made a substantial, direct, and intellectual contribution to the study, and approved it for publication.

## Conflict of Interest

The authors declare that the research was conducted in the absence of any commercial or financial relationships that could be construed as a potential conflict of interest.

## Publisher’s Note

All claims expressed in this article are solely those of the authors and do not necessarily represent those of their affiliated organizations, or those of the publisher, the editors and the reviewers. Any product that may be evaluated in this article, or claim that may be made by its manufacturer, is not guaranteed or endorsed by the publisher.
